# *De novo* transcriptome analysis of *Medicago falcata* reveals novel insights about the mechanisms underlying abiotic stress-responsive pathway

**DOI:** 10.1186/s12864-015-2019-x

**Published:** 2015-10-19

**Authors:** Zhenyan Miao, Wei Xu, Daofeng Li, Xiaona Hu, Jiaxing Liu, Rongxue Zhang, Zongyong Tong, Jiangli Dong, Zhen Su, Liwei Zhang, Min Sun, Wenjie Li, Zhenglin Du, Songnian Hu, Tao Wang

**Affiliations:** State Key Laboratory of Agrobiotechnology, College of Biological Sciences, China Agricultural University, Beijing, 100193 China; CAS Key Laboratory of Genome Sciences and Information, Beijing Institute of Genomics, Chinese Academy of Sciences, Beijing, 100029 China; State Key Laboratory of Plant Physiology and Biochemistry, College of Biological Sciences, China Agricultural University, Beijing, 100193 China; Present address: Department of Agronomy, Purdue University, West Lafayette, IN USA; Present address: Department of Genetics, Center for Genome Sciences and Systems Biology, Washington University School of Medicine, St. Louis, MO USA

**Keywords:** Medicago falcata, Transcriptome, Legume, Phytohormones, Nodule

## Abstract

**Background:**

The entire world is facing a deteriorating environment. Understanding the mechanisms underlying plant responses to external abiotic stresses is important for breeding stress-tolerant crops and herbages. Phytohormones play critical regulatory roles in plants in the response to external and internal cues to regulate growth and development. *Medicago falcata* is one of the stress-tolerant candidate leguminous species and is able to fix atmospheric nitrogen. This ability allows leguminous plants to grow in nitrogen deficient soils.

**Methods:**

We performed Illumina sequencing of cDNA prepared from abiotic stress treated M. falcata. Sequencedreads were assembled to provide a transcriptome resource. Transcripts were annotated using BLASTsearches against the NCBI non-redundant database and gene ontology definitions were assigned. Acomparison among the three abiotic stress treated samples was carried out. The expression of transcriptswas confirmed with qRT-PCR.

**Results:**

We present an abiotic stress-responsive M. falcata transcriptome using next-generation sequencing data from samples grown under standard, dehydration, high salinity, and cold conditions. We combined reads from all samples and de novo assembled 98,515 transcripts to build the M. falcata gene index. A comprehensive analysis of the transcriptome revealed abiotic stress-responsive mechanisms underlying the metabolism and core signalling components of major phytohormones. We identified nod factor signalling pathways during early symbiotic nodulation that are modified by abiotic stresses. Additionally, a global comparison of homology between the M. falcata and M. truncatula transcriptomes, along with five other leguminous species, revealed a high level of global sequence conservation within the family.

**Conclusions:**

M. falcata is shown to be a model candidate for studying abiotic stress-responsive mechanisms in legumes. This global gene expression analysis provides new insights into the biochemical and molecular mechanisms involved in the acclimation to abiotic stresses. Our data provides many gene candidates that might be used for herbage and crop breeding. Additionally, FalcataBase (http://bioinformatics.cau.edu.cn/falcata/) was built for storing these data.

**Electronic supplementary material:**

The online version of this article (doi:10.1186/s12864-015-2019-x) contains supplementary material, which is available to authorized users.

## Background

Abiotic stresses such as low temperature, high salinity, and drought adversely affect plant growth and productivity worldwide. The entire world is facing an increasing food requirement as the population grows continuously while crop and herbage productivity diminishes due to the deteriorating environment. Therefore, our understanding of the mechanisms underlying plant responses to external abiotic stresses is very important for breeding stress-tolerant crops and herbages, as well as sustaining the agricultural industry [1].

A variety of adaptive strategies to cope with abiotic stresses have evolved in plants. These include morphological, physiological, biochemical, and molecular responses [1–5]. Hundreds of plant genes are differentially regulated in response to abiotic stresses, as demonstrated by RNA-seq analyses. The complex network of regulatory genes necessary to sense and respond to abiotic stresses has been addressed. Regulatory component identified so far include transcription factors (TFs), plant hormones, noncoding RNAs, protein modifiers and epigenetic modifications [6–9].

The growth and development of all plants is determined by the interactions between their genome and growing environment. A multitude of complex signalling systems have evolved in plants to respond to external and internal cues to regulate growth and development under abiotic stresses. Phytohormones play critical regulatory roles in these processes. Generally, phytohormones control various developmental events throughout the plant life cycle, such as patterning, cell identity, and differentiation, as well as the coordinated growth of various reproductive organs [10]. In recent years, the metabolism and core signalling components of major phytohormones, such as abscisic acid (ABA), ethylene, jasmonic acid (JA), auxins, and gibberellins (GA), have been revealed [11]. In plants, the response to abiotic stresses is through the modulation of gene expression by phytohormone-mediated signalling processes. As summarised in recent reviews, ABA remains the best-studied hormone for plant stress responses [12–16]. However, other hormones, such as ethylene, JA, GA, and auxins, are being studied to a limited extent for abiotic stress responses [17–20].

Medicago falcata, which is closely related to alfalfa (Medicago sativa), is an economically and ecologically important legume herbage and is widely distributed throughout the world. M. falcata is especially attractive because it can be grown in adverse environment regions, such as infertile and sandy soils, and is, thus more tolerant against drought and cold compared with alfalfa [21, 22]. The combination of these traits makes M. falcata one of the best stress-tolerant candidate plant species. M. falcata also consists of numerous different genotypes, each of which has diverse growth and agricultural characteristics, indicating its agricultural importance [23]. A variety of genes were reported involved in tolerant mechanism of M. falcata. Expression of multiple genes encoding myo-inositol phosphate synthase (MIPS), galactinol synthase (GolS), early light induced protein (ELIP), hybrid proline rich protein (HyPRP), S-adenosylmethionine synthetase (SAMS), and a myo-inositol transporter-like (INTlike) in response to low temperature is also associated with cold tolerance in M. falcata plants [24–28]. Temperature-induced lipocalin (TILs) have initially been detected as massively increased proteins in response to low temperatures. Arabidopsis TIL1 is localized in the plasma membrane and its temperature response suggested a function in membrane stabilization during freezing stress [29]. A partial-length cDNA clone encoding a TIL has been obtained in M. falcata responsive to cold [30], implying its potential role in cold tolerance of M. falcata. As a legume species, M. falcata has the ability to fix atmospheric nitrogen through the establishment of a symbiotic association with bacteria called rhizobia, and this ability allows leguminous plants to grow in soils that are deficient in nitrogen, decreasing both the need for costly nitrogen fertilisers and the water pollution they may cause [31]. An organ, the root nodule, is formed to house this symbiosis. Within this organ, there is an exchange of nutrients: the bacteria provide the plant with ammonia, and the plant provides the bacteria with carbohydrates. Over the past several years, hundreds of plant and bacterial genes displaying differential expression during the nodulation process have been identified by transcriptome analyses in Medicago truncatula and Sinorhizobium meliloti [32–40], and many factors have been shown to control the nodulation process [41]. Studies of bacterial and plant mutants have led to the identification of plant genes involved in the early stages of symbiotic nodulation [42]. However, a transcriptomic analysis of nodulation signalling pathway gene expression in response to abiotic stresses is lacking in the literature.

The emergence of next-generation sequencing (NGS) technologies has allowed further study of species in which the genome sequence is available or previously unexplored species in re-sequencing and de novo sequencing aspects [43]. The technology, which allows the sequencing of the entire transcriptome (RNA-Seq), could provide expression profiles of either coding or non-coding RNAs, making important contributions to genome annotation [44, 45]. Transcriptome sequencing has become more and more popular in many research areas, especially for model species such as Arabidopsis [46], Cotton [47], Soybean [48], M. tuncatula [38, 39, 49–51] and non-model species, such as alfalfa [52–55], lentil [56], pea [57], pigeon pea [58] and cucurbits [59]. Although often used in species with a sequenced genome and high-quality gene predictions, the rapid development of software tools has allowed researchers to undertake the de novo assembly of a transcriptome based on only short sequence fragments, which has been widely applied in many plants [52, 60–63].

In this work, we present the expression profiles of M. falcata in plants grown under dehydration, high salinity, and cold conditions. We also explore evidence for novel abiotic stress-induced phytohormone metabolism and nodulation signalling. This is the first high-throughput transcriptomic study in stress-tolerant legume herbage, and we hope that it will be a valuable resource for breeding crops and herbages with enhanced abiotic stress-resistance abilities in legumes or other species.

## Results

### Evaluation of cold, dehydration and salt stresses response of three Medicago genotypes

In order to assess the resistance ability of M. falcata (PI502449), we test Medicago truncatula ecotypes A17 and R108 under different abiotic stresses, including cold, drought and salt stresses. The methods referred to Medicago Handbook [64] and was modified. Firstly, in the cold stress test, we find that the differences in mortalities (%) and root to aerial part ratios between M. falcata and M. truncatula are significant. After the treatment under −6 °C for 10 h, the mortality of PI502449 is 80 %, whereas the mortalities of A17 and R108 are both 100 %. Under −6 °C for 6 h, the mortalities of PI502449, A17, and R108 are 0 %, 85 %, and 100 %, respectively. Under −6 °C for 8 h, the mortalities of PI502449 is 20 %, the other two are 100 % (results are shown in Additional file 1D). Since the cold stress can inhibit the growth of roots, therefore, the root to aerial ratio, which is defined as the ratio between fresh weights of root to that of aerial part, can characterize the ability of alfalfa’s resistance to cold stress. The results show that, as the cold stress time becomes longer, the root of PI502449 can still grow in compare to that of R108 and A17 (Additional file 1A). Therefore, it is obvious that the PI502449 processes a stronger ability to resist the cold stress. Secondly, drought stress is applied 7 days (control), 10 days, 12 days and 14 days, respectively, followed by re-hydration, three replicates. Fresh weight and ratio of root to aerial part were measured. The results showed that the roots of PI502449 have relative higher water contents. PI502449 has the highest ratio of root to aerial part among R108, A17, and PI502449. To sum up, the M. falcata PI502449 has the strongest ability to resist drought. In the salt stress test, we treated the soil with 0 mM (Control), 100 mM, 150 mM and 200 mM NaCl, and then collect the content of soluble carbohydrate using a phenol-sulfuric acid method. The contents of soluble carbohydrate can be used as criteria for salt stress resistance, because the carbohydrate will increase under the salt stress to reduce the damage of NaCl to plantlet. Based on the results, we can conclude that A17 has the strongest ability to resist the salt stress, PI502449 the weakest (Additional file 1C). These results suggested that M. falcata PI502449 is more tolerant against drought and cold stresses compared with M. truncatula. Based on these results, we attend to reveal mechanisms involved in M. falcata response to abiotic stresses by using RNA-seq technology.

### RNA-seq using the Illumina platform and the de novo assembly of the transcriptome

In order to find out the early response genes of PI502449 under abiotic stresses, we analyzed the electrolyte leakage after the rapid stresses (Additional file 2). We picked the optimal stress treatment under which electrolyte leakage increased 2-fold or more compared with standard conditions for RNA-Seq. Plant materials were collected under dehydration (dehydrated for 2 h), high salinity (1 M NaCl for 2 h), or cold (0 °C for 8 h) treatment and then RNA were extracted. We developed complementary DNA (cDNA) libraries derived from M. falcata PI502449 grown under abiotic stress and standard conditions: dehydration stress plantlets (DS), high salinity stress plantlets (SS), cold stress plantlets (CS), and standard conditions (SC). Two libraries were constructed for one sample respectively, resulting in a total of eight cDNA libraries for the RNA-Seq analysis. A total of 33,432,584,900 nt of raw sequencing data were generated from the eight cDNA libraries. After a stringent quality filtration of the raw read data using the FASTX-toolkit (http://hannonlab.cshl.edu/fastx_toolkit/index.html), a total of 31,591,337,787 nt filtered data were selected for further analysis (Additional file 3).

To obtain a better assembly result, two assembly methods (single k-mer method and multiple k-mer method) were used for the de novo assembly of our sequencing data. The parameters used for the two methods are described in the Methods section. The de novo assembled transcripts were combined with 98,515 unique sequences (Additional file 4). The total base count of the assembly sequences was 84,416,200 nt, with the unique sequence lengths ranging from 200 to 14,802 nt. In order to explicitly evaluate the quality of the transcripts generated by assembly, we download 913 mRNAs already characterized in M. falcata from NCBI EST database. BLASTn comparisons were performed between assembly sequences and EST dataset. Total 869 (95.2 %) mRNAs had significant hits to assembly sequences. Putative functions for the assembly sequences were assigned using BLASTX searches against the non-redundant (nr) NCBI database, UniProt database, and M. truncatula proteome (for details, see the “Methods”; annotation files available at http://bioinformatics.cau.edu.cn/falcata). Putative functions were assigned for 50 % of the sequences. We also assigned gene ontology (GO) and Kyoto Encyclopedia of Genes and Genomes (KEGG) functional classes. Annotations for genes and pathways analysed in greater detail throughout this paper were manually curated to ensure accurate analysis and interpretation. In addition, we have pursued systemic co-expression/network analysis with our RNA-seq dataset using WGCNA software [65] and we integrated the results into FalcataBase (http://bioinformatics.cau.edu.cn/falcata/network/). Users can explore the players that contribute to cold, salt and dehydration response conveniently.

**Fig. 1:**
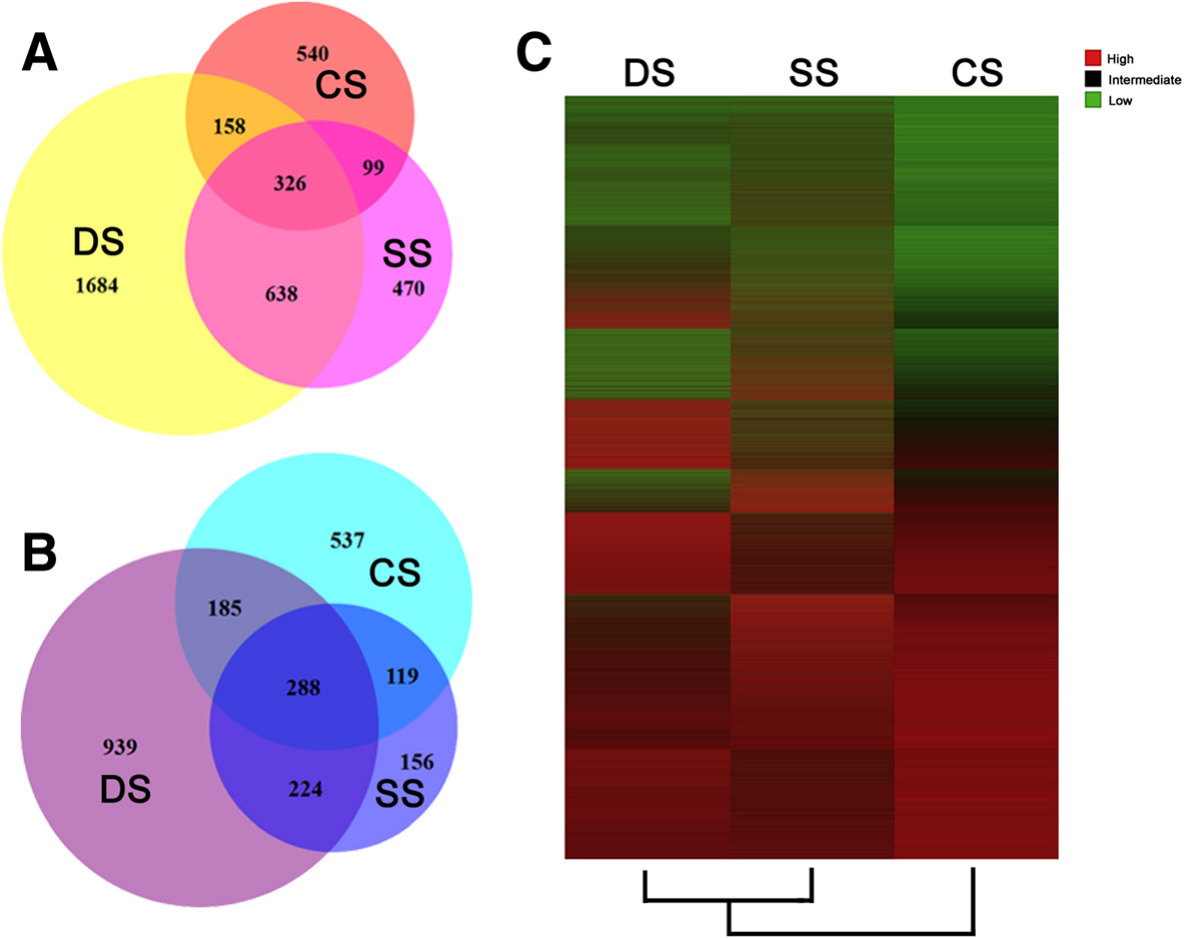
Transcripts differentially expressed due to abiotic stresses. A total of 6,158 transcripts were identified as differentially expressed in the DS, CS, and SS samples. To be considered differentially expressed, the transcript must have RPKM ≥ 2 in at least one library, a 2-fold or greater change between the stressed and standard condition, and P-value ≤ 0.05. **a** A total of 3,915 transcripts were up-regulated. **b** A total of 2,448 transcripts were down-regulated. **c** Heat map of the expression profiles of 6,158 transcripts differentially expressed due to abiotic stresses. Expression, represented by Z scores, is shown for transcripts differentially expressed due to three abiotic stresses. Red indicates high expression, black indicates intermediate expression, and green indicates low expression

The transcript profiles of M. falcata under dehydration, high salinity, and cold stresses were measured. We analysed the reads per kilobase per million reads (RPKM)-normalised gene expression counts for each sequence from eight samples. Previous studies have suggested that the expression of transcripts with low RPKM scores may be statistical artefacts [66, 67]. RPKM value depends by definition on the depth of the library. There are 20 million reads for a library on average. The mean read length is ~100 nt. Based on the depth of our sequencing libraries, the results indicated that a RPKM of 2 reflects a 4-fold coverage of the sequence (*coverage* = 2*RPKM* × 20*million* × 100*nt*/1000*bp*), assuming an equal distribution of reads across the sequence. Sequences with RPKM < 2 exhibited a low transcript abundance under the conditions tested and may not provide reliable expression data. Of the 98,515 sequences assembled, 52,725 had RPKM ≥ 2 in at least one sample and were considered transcriptionally active. A total of 6,158 transcripts were differentially expressed (2-fold change, P-value ≤ 0.05, RPKM ≥ 2) in response to at least one stress. Heatmap display of expression profile was shown in Fig. 1c. Overlapped transcripts between increased and decreased was also checked by Venn diagram analysis and shown in Fig. 1a and b.

We identified putative housekeeping genes that displayed little variation in expression but were expressed at relatively high levels (Additional file 5). To identify housekeeping genes, we first selected genes with an average RPKM-normalised transcript count greater than 10. We next selected the top 10 % of genes (938) with the lowest coefficient of variation (SD/mean) [48]. Finally, we picked up 587 single copy genes with the length more than 1000 nt. These housekeeping genes may be useful as reference genes in quantitative real-time PCR (qRT-PCR) or other experiments to normalise gene expression levels across different conditions [68].

Posttranscriptional regulation by microRNAs (miRNAs) is an important response to nutritional, biotic, and abiotic stresses [69, 70]. The sequencing of miRNAs requires special library construction. Therefore, the identification of mature miRNAs is beyond the scope of this study. However, transcripts regulated by miRNAs should contain sequences with almost perfect complementarity to known miRNAs. We used the psRNATarget programme [71] to screen the transcripts we identified as differentially expressed due to abiotic stresses for sequences potentially regulated by miRNAs using mature miRNAs from miRBase as queries. We identified 1,132 transcripts potentially regulated by 818 miRNAs (Additional file 6). Two of the 818 miRNAs identified in this analysis (miR166 and miR399) have been previously shown to directly regulate cold and salt responses [72]. Additionally, researchers have identified a number of miRNAs that are regulated by abiotic stresses [73–75]. Several of these miRNAs were also identified in this analysis.

### Differential expression of transcripts due to abiotic stresses

A total of 6,158 transcripts were differentially expressed in response to abiotic stresses: 4,460 genes in DS versus SC comparison, 2,356 genes in SS versus SC comparison, and 2,293 genes in CS versus SC comparison (Fig. 1; Additional file 7). Of the 4,442 differentially expressed transcripts identified in DS, 2,806 transcripts were up-regulated, whereas 1,636 were down-regulated. Of the 2,320 differentially expressed transcripts identified in SS, 1,533 transcripts were up-regulated, whereas 787 were down-regulated. Of the 2,252 differentially expressed transcripts identified in CS, 1,123 transcripts were up-regulated, whereas 1,129 were down-regulated. Additionally, 362 transcripts were induced and 288 transcripts were inhibited by all three stresses concurrently.

In response to abiotic stresses, the M. falcata transcriptome alters the expression of transcripts encoding for transcription factors (TFs). Of the 6,158 transcripts differentially expressed in response to all three abiotic stresses, 745 were identified as TFs (Table 1; Additional file 8). These transcripts belong to 65 TF families. The NAC and MYB-related TF families were the two largest families responding to the abiotic stresses identified in M. falcata. We identified 62 members of the NAC TF family exhibiting increased expression in DS, 33 members exhibiting increased expression in SS, and 12 members exhibiting increased expression in CS. The number of transcripts induced by cold stress was the smallest of the three stresses.

**Table 1:** TFs differentially expressed due to abiotic stresses

TF family	DS		SS		CS	
	Increase	Decrease	Increase	Decrease	Increase	Decrease
NAC	62		33		12	
MYB-related	30	19	17	9	16	12
bHLH	32	20	18	2	9	3
AP2-EREBP	26		15	6	15	10
WRKY	31	4	10	1	9	3
FAR1	19	8	6	2	6	3
MADS	24		10		3	
Orphans	25	2	10	1	7	
C2H2	13	3	11	3	4	5
C3H	15	4	10	2	1	
G2-like	4	12	3	2	2	4
Dof		6	8	7		3
GRAS	15		2	1	1	3
bZIP	10	2	5	3	1	5
SET	12	1	8		4	
PHD	11	2	1		1	1
Pseudo-ARR	7	2	2	2	2	
LBD	10		4		4	
ARF	6	2	3			

TFs with altered expression patterns between the abiotic-stressed samples and standard samples were used. The numbers of individual transcripts within a TF family exhibiting increased or decreased expression (a 2-fold or greater change) in the abiotic-stressed samples compared with the standard sample are shown

**Fig. 2:**
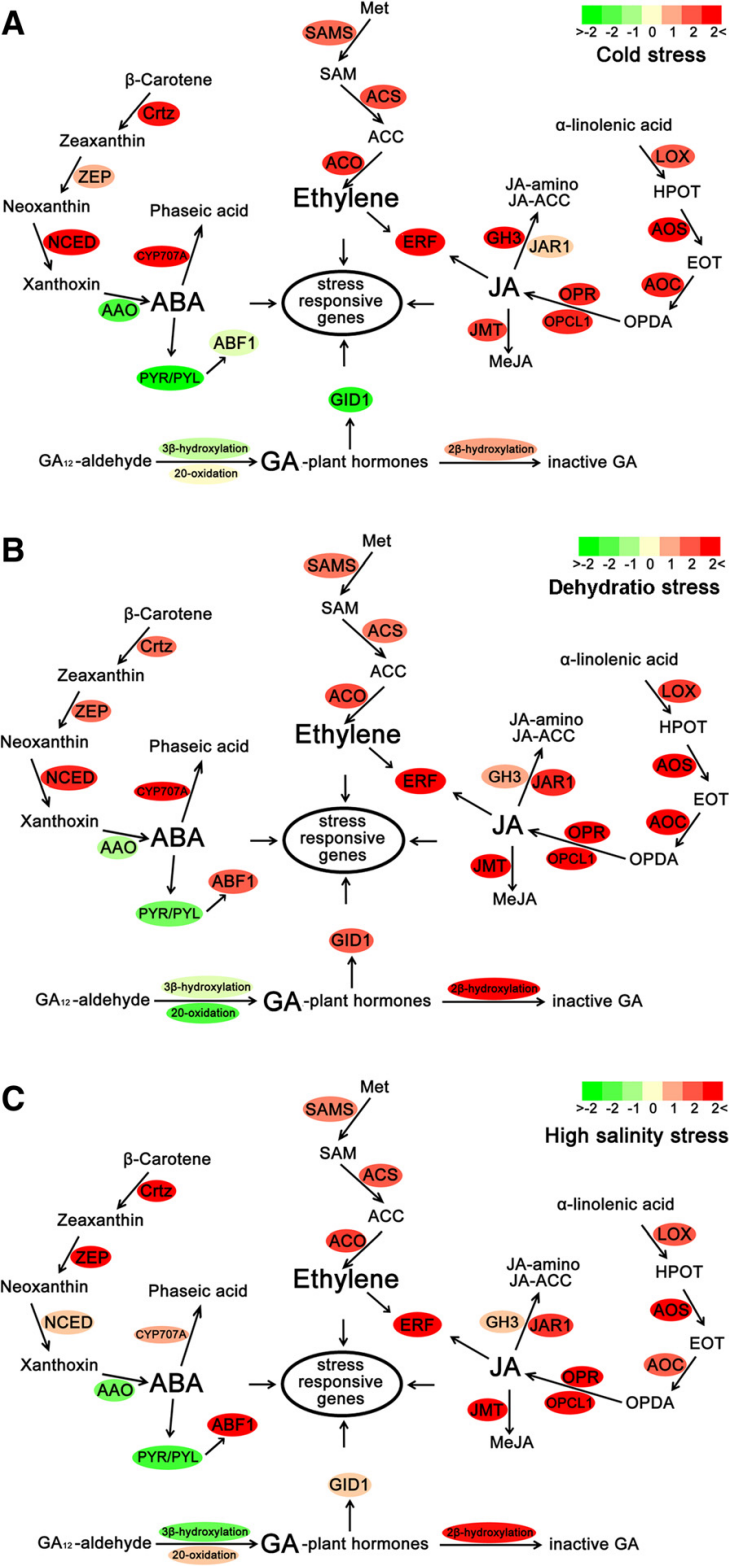
Expression and pathway distribution of phytohormone metabolism and signal transduction-related genes in *M. falcata*. **a** Expression pattern of key genes under cold stress. **b** Expression pattern of key genes under dehydration stress. **c** Expression pattern of key genes under high salinity stress. Additional abbreviations are as follows: Crtz (beta-carotene 3-hydroxylase); ZEP (zeaxanthin epoxidase); NCED (9-cis-epoxycarotenoid dioxygenase); AAO (abscisic-aldehyde oxidase); PYR/PYL (PYRABACTIN RESISTANCE1 / PYRABACTIN RESISTANCE 1-LIKE); ABF1 (ABRE-binding factors 1); GID1 (GA-INSENSITIVE DWARF1); SAMS (*S*-adenosylmethionine synthetase); ACS (1-aminocyclopropane-1-carboxylic acidsynthetase); ACO (1-aminocyclopropane-1-carboxylic acid oxidase); ERF (ethylene response factor); LOX (lipoxygenase); AOS (allene oxide synthase); AOC (allene oxide cyclase); OPR (12-oxo-phytodienoic acid reductase); OPCL1 (OPC-8:0 CoA ligase 1); JMT (jasmonate O-methyltransferase); and JAR1 (Jasmonate response 1). Filled colours correspond with their degree of regulation by the stresses

As noted in previous studies, abiotic stress frequently influences phytohormone metabolism and signal transduction. Meanwhile, phytohormones play critical roles in responding to external and internal cues to regulate plant growth and development under abiotic stresses [10]. To further understand how M. falcata acclimates to abiotic stresses, we evaluated our transcriptome data for differential expression genes to phytohormone metabolism and signal transduction, including abscisic acid (ABA), ethylene, jasmonic acid (JA), and gibberellins (GA) (Fig. 2).

ABA signalling and its key role in the adaptation of plants to stress conditions has been the focus of many studies since the early 1990s [76]. Thus, we queried the database for differentially expressed transcripts related to ABA biosynthesis enzymes, such as Crtz (beta-carotene 3-hydroxylase), ZEP (zeaxanthin epoxidase), and NCED (9-cis-epoxycarotenoid dioxygenase), as well as AAO (abscisic-aldehyde oxidase) and ABA catabolism enzymes, such as CYP707A (Fig. 2). As expected, most of the transcripts for the ABA biosynthesis enzyme genes displayed enhanced expression in DS, SS, and CS. However, transcripts for the ABA catabolism enzyme genes showed decreased expression in DS, SS, and CS. Unexpectedly, transcripts for the AAO gene, which encodes for an enzyme that catalyses the final step of ABA biosynthesis, showed different levels of decreased expression in all stress-treated samples. Meanwhile, we determined the content of ABA after cold treatment. Compared with M. truncatula, the content of ABA in M. falcata dropped significantly (Additional file 9). Furthermore, PYR/PYL (Pyrabactin Resistance 1/Pyrabactin Resistance 1-Like) genes, identified as ABA receptors, showed obvious decreased expression in DS, SS, and CS. ABF transcription factors are the key regulators of ABA signalling. Interestingly, transcripts for a member of this family, named ABF1, and exhibiting differential expression were found; up-regulated expression was observed in the DS and SS samples, while down-regulated expression was observed in the CS samples. Together with ABA, gibberellins (GAs) act as a main regulating factor in seed germination; however, their role is antagonistic compared with ABA. A principal pathway for GA plant hormones can be drawn from GA_12_-aldehyde. A stepwise analysis of GA metabolism revealed the conversion of GA_12_-aldehyde to bioactive GA_4_ and inactive GA_34_ [77]. In our transcriptome data, the GA 20-oxidase and 3β-hydroxylase transcripts showed different levels of decreased expression in all stress-treated samples; however, the 2β-hydroxylase transcripts displayed the respective increased expression in these samples (Fig. 2). Interestingly, we identified some transcripts for a GA receptor named GID1 (GA-INSENSITIVE DWARF1) exhibiting differential expression. Compared with the down-regulated expression in the CS sample, these transcripts showed up-regulated expression in the DS and SS samples.

Our transcriptome data demonstrated that both ethylene and JA metabolism are altered under abiotic stress conditions. The biochemistry of ethylene biosynthesis has been a subject of intensive study in plant hormone physiology [78]. Major breakthroughs in the ethylene synthesis pathway were the establishment of S-adenosylmethionine (SAM) and 1-aminocyclopropane-1-carboxylic acid (ACC) as the precursors of ethylene. Based on this knowledge, we identified differentially expressed transcripts related to ethylene biosynthesis enzymes (Fig. 2). All of the transcripts for ethylene biosynthesis enzyme genes showed enhanced expression in the DS, SS, and CS samples. In addition, jasmonates are also important regulators in plant responses to abiotic stresses and in development [79]. The expression of the transcripts involved in JA biosynthesis was increased in the DS, SS, and CS samples (Fig. 2). Upon further analysis, we noted that all three abiotic stresses increased the expression of transcripts related to the JA metabolic pathway, which would contribute to the increased formation of the bioactive JA compound. Additionally, ethylene response factor (ERF) is an upstream component in both JA and ethylene signalling and is involved in abiotic stress resistance [80]. The transcripts for the ERF genes exhibited increased expression in DS, SS, and CS. A reconstruction of metabolic pathways based upon the expression profiles of transcripts expressed during phytohormone biosynthesis provides evidence for previously unrecognised abiotic stress-induced pathways and reinforces the interpretation that phytohormone metabolism is modified under abiotic stress conditions. The expression of transcripts involved in phytohormone metabolism and signal transduction was confirmed with qRT-PCR (Table 2).

**Table 2:** Confirmation of the RNA-Seq expression profiles with qRT-PCR

Transcript	RNA-Seq(CS)	RNA-Seq(DS)	RNA-Seq(SS)	qRT-PCR(CS)	qRT-PCR(DS)	qRT-PCR(SS)	Annotation
Mf53179	5.93	0.69	0.05	9.28	5.01	2.43	DREB protein
Mf40807	3.81	1.30	0.91	4.27	4.06	1.71	GH3
Mf52262	2.79	3.45	2.16	7.24	8.87	5.47	NFR5
Mf44686	2.55	6.14	2.35	7.62	7.03	3.19	ERF
Mf24843	2.45	5.53	3.08	7.28	9.46	5.09	ERF
Mf94524	2.22	3.83	0.66	4.43	3.25	0.45	CYP707A
Mf21376	2.18	1.70	0.40	2.13	1.57	0.33	NCED
Mf89827	2.17	4.92	5.37	1.74	6.17	5.14	Stress-induced receptor
Mf26361	2.11	2.72	1.95	1.84	4.19	0.43	OPCL1
Mf20359	2.04	0.58	1.34	0.15	4.39	2.06	ACS
Mf40505	1.93	3.50	2.66	2.01	5.98	1.40	OPR
Mf51793	1.88	6.03	3.99	5.03	6.35	4.40	Calmodulin binding
Mf23563	1.86	1.12	2.39	1.86	1.12	2.40	CrtZ
Mf84393	1.49	3.81	2.09	3.76	4.96	3.95	JMT
Mf42223	1.48	3.83	1.88	4.74	4.71	1.66	AOS
Mf34249	1.40	1.88	1.29	3.81	1.02	1.43	ACS
Mf84453	1.28	1.53	1.31	−0.16	1.05	−2.60	LOX
Mf31223	1.26	1.52	1.24	1.74	3.19	0.05	AOC
Mf37052	1.24	3.75	3.10	2.81	2.83	1.09	NFR1
Mf19438	1.21	2.24	1.65	5.07	3.66	1.07	SAMS
Mf21208	1.16	1.68	1.12	0.70	3.28	1.34	2-beta-dioxygenase
Mf37096	1.15	4.29	2.69	1.03	3.22	0.23	DMI3
Mf46196	1.09	4.29	5.05	3.56	10.67	9.19	NCED
Mf41585	1.08	1.51	1.14	0.10	1.95	−1.19	SNF1
Mf84582	0.96	−1.01	0.29	−0.52	−2.24	−2.72	cytochrome P450
Mf19924	0.96	3.13	1.70	1.18	0.89	0.64	SAMS
Mf35541	0.83	2.21	1.65	0.26	1.46	−1.00	SNF1
Mf53098	0.75	1.69	1.60	0.32	1.67	0.43	JAR1
Mf52729	0.70	−0.38	0.46	5.19	−0.63	3.87	Cysteine-rich receptor
Mf29322	0.65	0.91	1.75	1.69	1.25	1.20	ACO
Mf32232	0.59	1.13	3.02	−0.04	1.13	3.01	ZEP
Mf46161	0.49	3.69	1.20	2.24	3.79	0.46	ACS
Mf33973	0.42	1.21	0.25	2.01	1.73	0.69	DMI1
Mf22267	0.18	0.45	−0.53	0.43	0.40	−0.87	CrtZ
Mf37546	0.15	1.34	0.78	0.30	0.25	−0.61	JAR1
Mf24274	0.14	−0.68	−0.40	0.07	−1.38	−0.26	20-oxidase
Mf58069	0.11	0.48	1.82	1.03	0.17	1.15	ACO
Mf88132	−0.21	1.26	2.37	−0.66	2.33	2.90	ABF 1
Mf46174	−0.25	−1.17	−0.25	−1.11	−0.95	−0.41	ZEP
Mf23784	−0.77	−0.72	−1.00	−1.93	−0.06	−0.42	NSP11
Mf49154	−0.95	0.58	1.87	−0.56	2.59	3.75	Importin
Mf50824	−0.96	3.51	1.26	−0.79	1.20	0.70	DMI2
Mf54137	−1.11	1.41	0.18	−3.07	5.33	3.96	Nodule receptor kinase
Mf22751	−1.33	−1.76	−0.74	−0.46	−0.73	−1.25	PYR/PYL
Mf11674	−1.36	−2.59	−0.26	−0.68	−1.45	−2.58	Leghemoglobin
Mf24497	−1.49	−0.88	−1.62	−3.93	−0.23	−2.52	ENOD
Mf34644	−1.54	0.43	0.14	−0.55	0.99	0.60	GID1
Mf20666	−1.76	1.26	0.37	−0.16	0.92	0.20	GID1
Mf56011	−1.77	−1.09	−0.47	−2.17	−2.48	−1.62	AAO
Mf38812	−2.21	2.13	2.99	0.52	0.68	0.72	DMI3
Mf17441	−2.26	−1.66	−2.11	−1.45	−0.71	−1.14	AAO
Mf87623	−2.45	−1.08	−1.48	−1.04	−0.35	−2.13	PYR/PYL
Mf49788	−3.16	−2.17	−2.18	−0.70	−0.57	−2.60	NSP2
Mf20500	−3.78	3.32	−0.57	−1.98	5.88	−2.94	ERN2
Mf21830	−4.11	−1.75	−2.31	−1.84	−0.73	−4.88	ENOD
Mf29128	−5.20	1.10	−0.12	−0.46	−0.05	−1.69	3-beta-hydroxylase

The expression levels of CS, DS, SS, and SC were calculated, and pairwise comparisons (CS versus SC, DS versus SC, and SS versus SC) were calculated. Expression ratios for the RNA-Seq data, shown as the fold change, were calculated for cold [RNA-Seq (CS)], dehydration [RNA-Seq (DS)], and high salinity [RNA-Seq (SS)] using the DESeq programme. Ratios for qPCR analyses were calculated for cold [qRT-PCR (CS)], dehydration [qRT-PCR (DS)], and high salinity [qRT-PCR (SS)] using a previously described method [114]. All expression ratios are presented as log_2_(treatment/standard). Negative values indicate that the standard has a higher expression level than the treatment, and expression ratios of 0 indicate no difference in expression between the treatment and standard

**Fig. 3:**
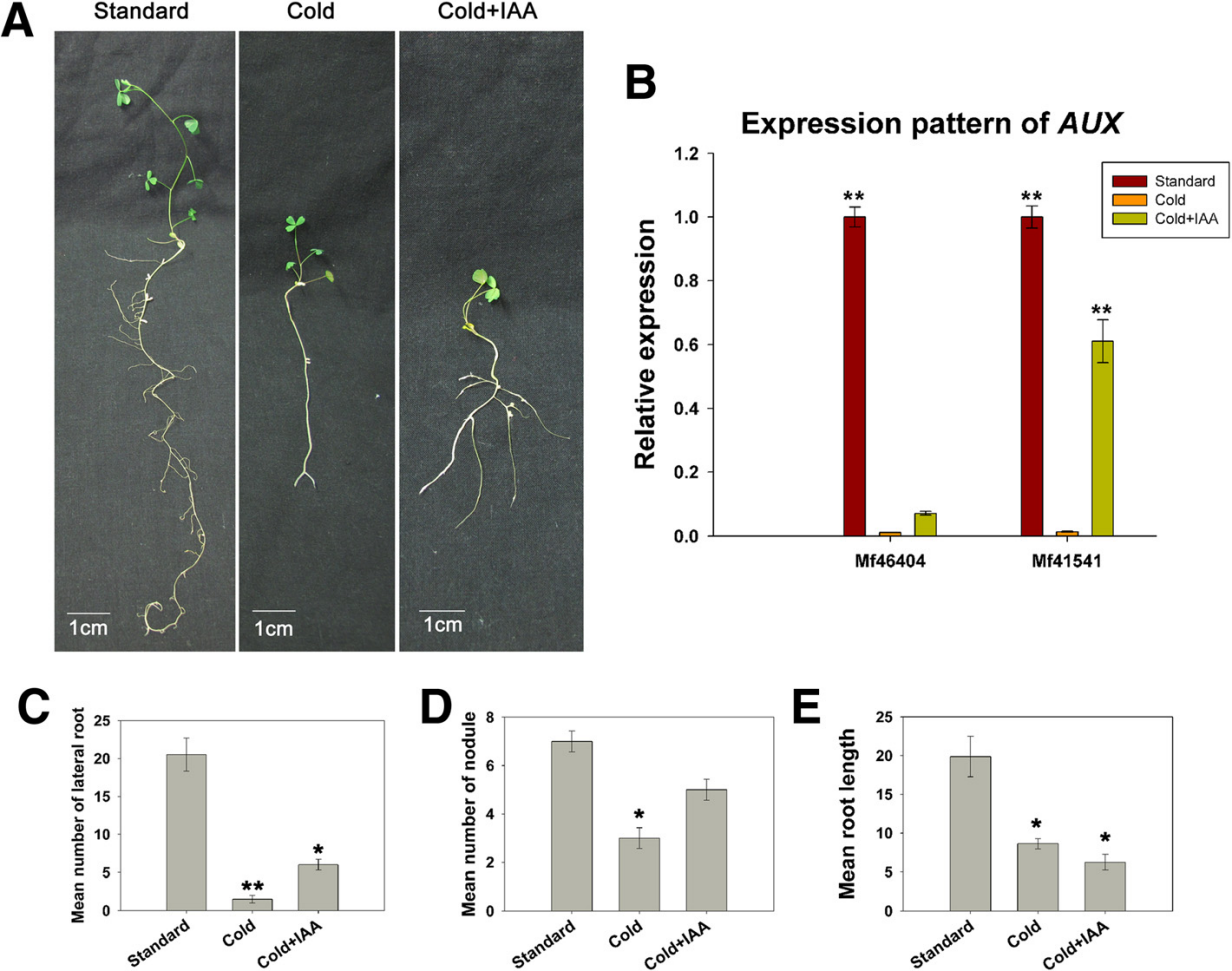
Auxin application influences cold stress-induced root development. **a** Standard condition, cold treatment, and cold treatment with IAA application phenotypes of *M. falcata*. **b** Relative expression level of genes coding for the auxin transporter (AUX). **c** Number of lateral roots under standard conditions, cold treatment, and cold treatment with IAA application. The mean values and SD were calculated from the results of the three replicated experiments. (Student’s *t*-test, **P < 0.001, *P < 0.01). **d** Number of nodules under standard conditions, cold treatment, and cold treatment with IAA application. The mean values and SD were calculated from the results of three replicated experiments. (Student’s *t*-test, **P < 0.001, *P < 0.01). **e** Length of root under standard conditions, cold treatment, and cold treatment with IAA application. The mean values and SD were calculated from the results of the three replicated experiments. (Student’s *t*-test, **P < 0.001, *P < 0.01)

Early reports suggested that auxin might affect plant stress response and abiotic stresses might regulate the expression of auxin-responsive genes [17, 81]. In our transcriptome data, few transcripts related to auxin biosynthesis showed differential expression. Interestingly, an auxin-responsive gene named GH3 was indentified up-regulated almost 16-fold in CS. To better understand the role of auxins in plant responses to cold stress and in root development, we treated plants with either indoleacetic acid (IAA) or cold stress (Fig. 3). The application of cold stress led to a dramatic reduction of lateral roots, nodule number, and root length. By comparison, the addition of IAA had no effect on root length; however, the number of lateral roots and nodules increased. To further investigate the mechanism underlying auxins in plant responses to cold stress and in root development, we used qRT-PCR to measure the expression levels of genes involved in auxin transport. Not surprisingly, cold application appeared to inhibit the expression of AUX, an important gene in auxin transport. The expression of AUX increased after the application of exogenous IAA. Overall, these qRT-PCR results illustrate the importance of auxin transport in M. falcata responses to cold stress and in root development.

**Fig. 4:**
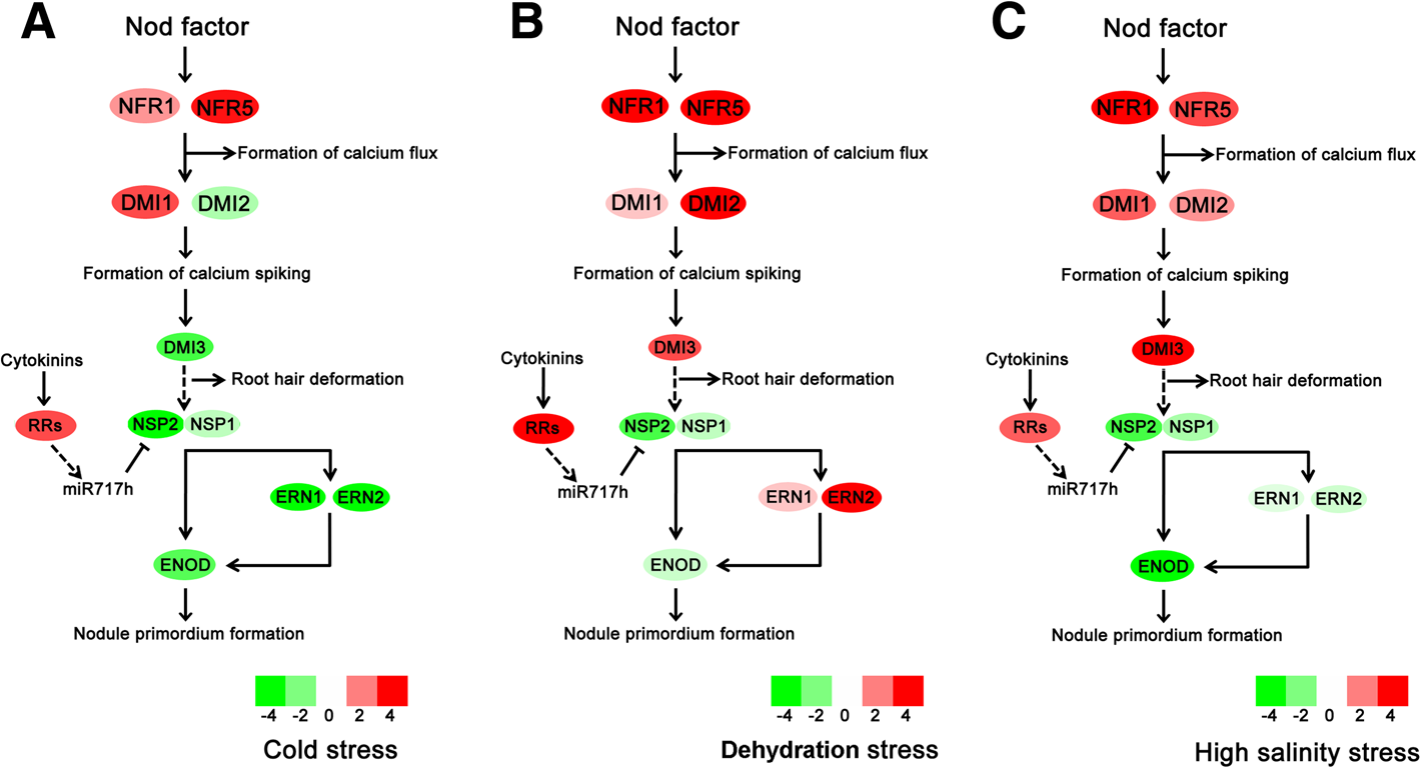
Expression pattern of genes involved in early symbiotic nodulation. **a** Expression pattern of key genes under cold stress. **b** Expression pattern of key genes under dehydration stress. **c** Expression pattern of key genes under high salinity stress. Additional abbreviations are as follows: NFR (nod factor receptor); DMI (does not make infections); NSP (Nodulation signalling pathway); ERN (ERF required for nodulation); and ENOD (early nodule). Filled colours correspond with their degree of regulation by the stresses

Nodules are legume-specific organs that are formed to fix atmospheric nitrogen through the establishment of a symbiotic association with bacteria known as rhizobia. To investigate the effect of abiotic stresses on nodule formation, we identified differentially expressed transcripts related to nod factor signalling pathways during early symbiotic nodulation (Fig. 4). NFR1 and NFR5 have been recognised as nod factor receptors in a previous study [82]. Transcripts for the NFR1 and NFR5 genes showed different levels of increased expression in all stress-treated samples. DMI1 and DMI2 are two components of the nod factor signalling pathway upstream of calcium spiking. DMI3 is a component of the nod factor signalling pathway downstream of calcium spiking and is responsible for the conversion of the calcium spiking signal. In the DS and SS samples, the transcripts for the DMI1, DMI2, and DMI3 genes displayed similar increased expression levels. In the CS sample, the transcripts for the DMI1 gene exhibited up-regulated expression. In contrast, the transcripts for the DMI2 and DMI3 genes exhibited down-regulated expression. ENOD11 is widely used as an early infection-related molecular marker for endosymbiotic associations involving both rhizobia and arbuscular mycorrhizal fungi [83]. Enod11 can be induced by several transcriptional regulators, including NSP1, NSP2, ERN1, and ERN2. The transcripts for the Enod11 gene exhibited obviously decreased expression in the DS, SS, and CS samples. In our transcriptome data, NSP1 and NSP2 displayed different levels of decreased expression in all stress-treated samples. ERN1 and ERN2 exhibited differential expression; up-regulation was found in the DS samples, while down-regulation was observed in the SS and CS samples. RRs (response regulators) act upstream of the NSP pathway to regulate the cytokinin response and nodule organogenesis [84]. The transcripts for the RRs genes exhibited obvious increased expression in the DS, SS, and CS samples. The expression level of the transcripts involved in the nod factor signalling pathways during early symbiotic nodulation was confirmed with qRT-PCR (Table 2).

### RNA-Seq expression validation by qRT-PCR

Transcripts known to be involved in the abiotic stress response (such as dehydration, high salinity, and cold) were confirmed by qRT-PCR to have expression patterns similar to those measured by RNA-Seq. Additionally, the expression profiles of transcripts involved in phytohormone metabolism and symbiotic nodulation signal transduction were also measured by qRT-PCR (Additional files 10 and 11). Finally, transcripts involved in auxin transport were identified in the assembly and used to design primers for qRT-PCR analysis to measure the expression profiles in plants treated with IAA. In total, 56 transcripts were evaluated by qRT-PCR, with 95.2 % consistent with the RNA-Seq data (Table 2).

### Comparative transcriptomics of M. falcata and with the other legume species

**Fig. 5:**
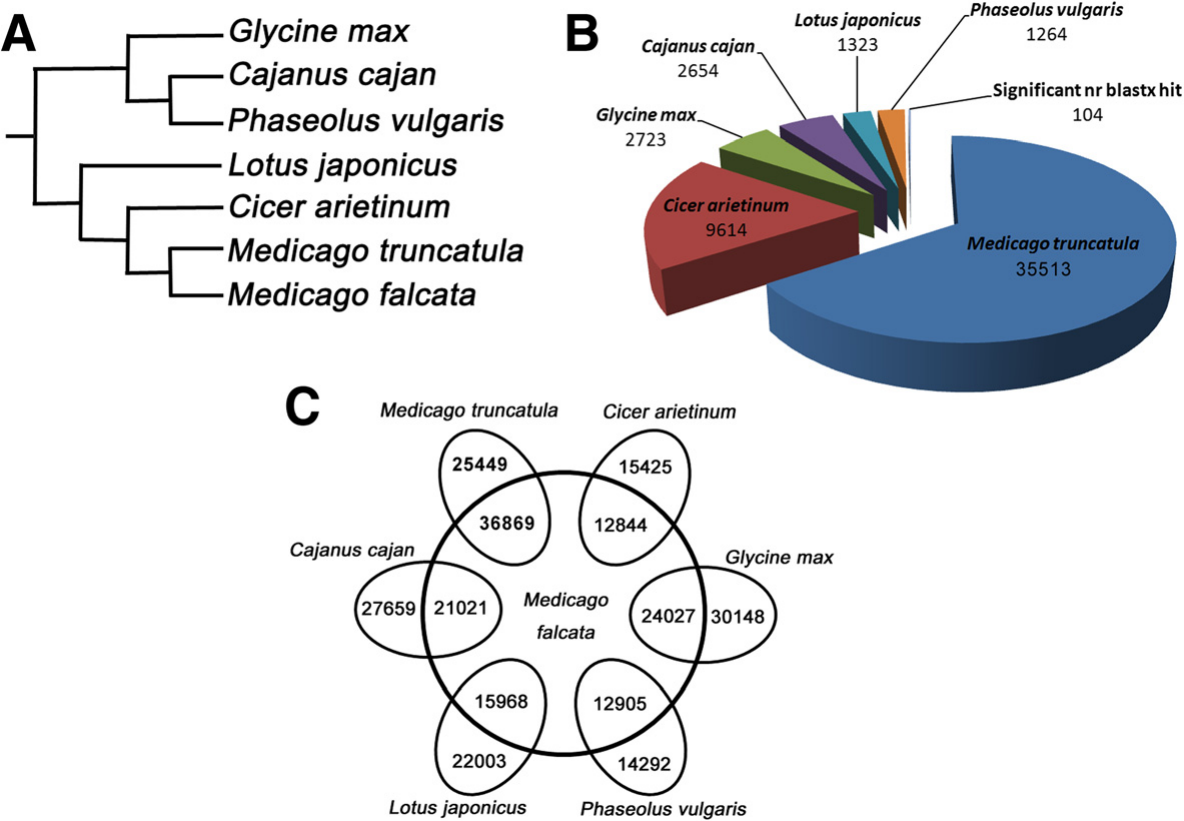
Comparative transcriptomics of *M. falcata* with six leguminous species. **a** Representation of the legume phylogeny. **b** BLASTx comparison of the *M. falcata* transcriptome assembly with *M. truncatula*, *C. arietinum*, *G. max*, *C. cajan*, *L. japonicus*, and *P. vulgaris*. The top BLAST hit (e ≤ 10–5) for each *M. falcata* transcript to the six species is shown. Contigs without significant hits were then compared with the NCBI peptide non-redundant (nr) database. **c** Six pairwise tBLASTn comparisons of legume species to the *M. falcata* transcriptome assembly. Sequences with significant homology (e ≤ 10–5 and positive percent ≥ 70 %) shared between the six legume species and *M. falcata* are shown on the inner circle

Previous phylogenetic analyses of the legumes have divided the family into seven major clades: Cladrastis, genistoidsensulato, dalbergioidsensulato, mirbelioid, millettioid, robinioid, and inverted-repeat-lacking (IRLC) [85]. Medicago truncatula is a member of the IRLC clade and is closely related to M. falcata (Fig. 5a). To explore the relationship between M. falcata and other legumes at the transcriptome level, we compared the assembled and translated M. falcata transcriptome to a peptide database derived from the sequenced genomes of Medicago truncatula, Cicer arietinum, Lotus japonicas, Glycine max, Phaseolus vulgaris, and Cajanus cajan. A BLASTx comparison of the M. falcata transcriptome to this peptide database showed that 35,513 M. falcata contigs had significant (e ≤ 1e^−5^) top hits to M. truncatula (Fig. 5b). C.arietinum had the next highest number of top hits (9,614). A BLASTx comparison of the remaining sequences without significant hits to one of the six legume species revealed that 104 sequences had significant hits in the NCBI non-redundant (nr) peptide database.

To examine the degree of conservation between M. falcata and other sequenced legume species, six pairwise tBLASTn comparisons were performed between M. falcata and each of the six legume species (Fig. 5c). M. truncatula had the highest number of sequences with significant hits to the M. falcata database (e ≤ 1e^−5^ and ≥70 % positive match percent), and the greatest proportion of peptides with significant matches (36,869/62,318). All six species had at least 42 % of their proteins significantly represented in the M. falcata database. A global view of the top M. falcata transcripts and their similarity to each M. truncatula peptide (primary transcripts only) is shown in Additional file 12C. Of the M. truncatula loci, 6,743 of 62,318 had transcripts with >70 % similarity and >70 % coverage in the M. falcata transcriptome.

To more closely examine the level of global sequence conservation between M. falcata and M. truncatula, we further examined a BLASTx comparison of the M. falcata transcriptome assembly with the M. truncatula peptide database (primary transcripts only). The relative homology of each predicted peptide to the most similar M. truncatula protein was measured by the percent of positive sequence similarity (Additional file 12A) and percent coverage (Additional file 12B). A smooth scatter plot representing the percent similarity and percent coverage for each M. falcata sequence compared with the closest M. truncatula peptide sequence is shown in Additional file 12C. A proportion (>40 %) of transcripts possessed at least 70 % similarity to a M. truncatula protein. A total of 13,538 M. falcata transcripts had at least one match to a M. truncatula gene, with >70 % similarity/>70 % coverage. Of the M. falcata transcripts, 1,454 had ≥95 % similarity and coverage, 2,029 transcripts were between 80-95 % similarity and coverage, 730 transcripts were between 70–80 % similarity and coverage, 7,458 transcripts were < 70 % similarity and coverage, and 49,400 transcripts lacked a significant BLASTx hit (e ≤ 1e^−5^) to a M. truncatula peptide.

## Discussion

Several studies utilising microarrays and RNA-Seq have documented the responses of plants to abiotic stresses. In this study, we extended our fundamental understanding of plant acclimation to abiotic stresses through an RNA-Seq whole transcriptome analysis of dehydration, high salinity, and cold treatment in M. falcata. Although our analysis primarily focused on phytohormone metabolism and signalling, we also explored the effect of abiotic stresses on nod factor signalling pathways during early symbiotic nodulation. Our data provide the foundation for what to our knowledge is the first M. falcata gene index (MFGI). We report 98,515 unique sequences, of which more than 6,000 respond to abiotic stresses. Moreover, we report previously unrecognised transcriptional responses to abiotic stresses.

### Comparing the M.falcata transcriptome with other legume transcriptomes

We sequenced, assembled, and annotated the M. falcata transcriptome. The draft transcriptome consists of 98,515 unique sequences, of which 51,040 were annotated and the functions of the rest transcripts were unknown. Of these transcripts, 34.5 % were most similar to M. truncatula genes, and 52 % had top hits in the legume, indicating a high level of sequence conservation across the family. BLAST comparisons between M. falcata and six other sequenced legume species showed that our M. falcata transcriptome has good coverage of homologous sequences. Compared with the other species, M. truncatula had the largest proportion of peptides with significant matches (36,869/62,318). In total, all of these six species had 107,779 proteins significantly represented in the M. falcata database. We inferred that multiple sequences in the M. falcata assembly may divide from one single protein-coding transcript. These analyses are consistent with previous phylogenetic findings that M. falcata is more closely related to M. truncatula than other species [85]. The M. falcata transcriptome will provide evidence for the expression of predicted genes in the M. truncatula genome.

### Genes commonly expressed under abiotic stresses

We identified 166 transcripts containing sequences homologous to 71 identified helicase proteins in other plants. Nine transcripts were induced by all three stresses. These transcripts were homologous to seven helicase proteins in plants, including Ricinus communis, Vitis vinifera, and Arabidopsis lyrata, indicating that at least seven helicase genes in M.falcata could be induced to respond to dehydration, high salinity, and cold. The helicase involved in the abiotic stress response was first identified in the legume pea [86]. In the pea, DNA helicase 47 (PDH47, GenBank accession number: AAN74636.1) was only induced by salt and cold stress and not by dehydration stress. This induction was also tissue-specific and regulated by responses to heat and ABA stress. In our data, three transcripts were similar to a DEAD-box ATP-dependent RNA helicase (GenBank accession number: XP_002527090.1), and one transcript was similar to a RNA helicase (GenBank accession number: AAF40306.1). The importance of RNA helicases in stress responses was also reviewed in a previous research study [87]. An increased expression level was also observed in genes encoding for RNA polymerases. Eight transcripts corresponding to eight proteins from four species were induced by all three stresses and accounted for 2.8 % of all of the polymerase transcripts identified. The relationship between poly (ADP-ribose) polymerase (PARP) and abiotic stresses has been studied in the soybean, and this enzyme was found to mediate DNA repair and programmed cell death processes during responses to mild and severe stresses, respectively [88]. In addition, PARP inactivation has been reported to increase the tolerance of plants against a broad range of abiotic stresses by inhibiting the processes of cell death and reducing energy consumption [89]. Choline, an important precursor of the membrane phospholipid phosphatidylcholine, is used to produce glycine betaine, which functions as an osmoprotectant conferring salt, drought, and other stress tolerance in plants [90, 91]. Phosphoethanolamine N-methyltransferase (PEAMT, EC 2.1.1.103) is believed to be a key enzyme of the choline biosynthesis pathway. Note that two transcripts with sequences homologous to a putative PEAMT protein from R. communis were up-regulated by all three stresses.

### TF expression is altered by abiotic stresses

Interestingly, all the major TF families induced by abiotic stresses are directly related to either a general stress response (NAC, MYB, bHLH, and WORKY) or a specific hormone pathway (AP2-EREBP, ARF, and Pseudo-ARR). The largest class of TFs induced by abiotic stresses in the three stressed samples was the NAC TF family. A total of 62, 33, and 12 NACs were up-regulated due to dehydration, high salinity, and cold stress, respectively. NAC is regarded as a plant-specific transcription factor family member and expressed in many tissues and during many developmental stages [92]. A further systemic analysis of the NAC transcription factor family at the genome-wide level reported that approximately 30 % of members of the NAC family are up-regulated during abiotic stress in rice, with 11 members induced by at least three types of abiotic stresses [93]. Additionally, in our data, the largest class of TFs induced by cold stress was the MYB TF family. A total of 16 MYBs were up-regulated by cold stress, suggesting that this TF family plays a major role in cold tolerance. A R2R3-type MYB has been identified as a master regulator of the cold stress response [94], and these results are consistent with our RNA-Seq data. Interestingly, in our transcriptome data, lower numbers of TFs were up-regulated by cold stress than the other two stresses, potentially because the response to cold stress appears to be less active than dehydration and salt stresses. In our opinion, because M. falcata was originally grown in extremely cold environments, the cold-responsive genes were already expressed at a higher level. This hypothesis was inspired by a study in Arabidopsis and salt cress, which illustrated that the stress tolerance of salt cress was due to stress-inducible genes that were constitutively overexpressed in salt cress but inducible in Arabidopsis [95].

### Abiotic stresses modify phytohormone metabolism and signal transduction

Previous studies have demonstrated that abiotic stresses have a striking effect on phytohormone metabolism and signalling. Transcriptomic and metabolomic analyses have demonstrated increased ABA, ethylene, and JA metabolism in several plants [12–20]. We not only reconfirm these initial findings but also extend our understanding of modified phytohormone metabolism in plants. The process from stress signal perception and the trigger of ABA biosynthesis to the dynamic regulation of the ABA level is an important stress signalling pathway in cells. Our data demonstrate the enhanced transcript expression involved in most steps of ABA biosynthesis, including transcripts for Crtz, ZEP, and NCED. The enhanced expression of ABA biosynthesis pathway genes may lead to increased endogenous ABA. Interestingly, transcripts for the AAO gene, which encodes for an enzyme that catalyses the final step of ABA biosynthesis, showed decreased expression in all stress-treated samples. Meanwhile, transcripts for the CYP707A gene, which encodes for an enzyme that catalyses ABA catabolism, showed increased expression in all stress-treated samples. Together with the ABA content determined under cold stress, we hypothesise that increased endogenous ABA may inhibit ABA biosynthesis and advance ABA catabolism to some extent, which is aimed at the dynamic regulation of the ABA level and maintaining the ABA level in balance. ABA perception through PYR/PYL receptors is required for the basal ABA signalling that promotes plant growth and normal seed production and that regulates steady state transpiration [96]. The expression of transcripts for PYR/PYL genes was inhibited by all three stressed samples. Compared with cold stress, the dehydration and high salinity stresses both enhanced the expression of the ABF1 genes observably and induced the expression of ABA-responsive genes further. In contrast, compared with enhancing ABA biosynthesis, all three abiotic stresses inhibited the conversion of GA_12_-aldehyde to bioactive GA_4_ and advanced the conversion of bioactive GA_4_ to inactive GA_34_, indicating that GA plays an antagonistic role in response to abiotic stresses in M. falcata compared with ABA. Additionally, the antagonistic role of GA is further confirmed by the expression pattern of GA receptors, namely GID1, being contrary to ABA receptors in the DS and SS samples.

The process from stress signal perception to the dynamic regulation of ethylene and JA metabolism is an important abiotic stress signalling pathway in plants. Compared with the downstream events in ethylene and JA signal transduction, the studies in this field are relatively lagging. Our discovery sheds new light on abiotic stress-induced ethylene and JA metabolic responses. We found evidence for pathways that lead to enhanced ethylene and JA biosynthesis. In our transcriptome data, we detected three key enzymes related to the ethylene biosynthesis pathway. All of the transcripts for ethylene biosynthesis enzyme genes showed enhanced expression in all three stressed samples. Consistent with ethylene, JA biosynthesis-related transcripts exhibited similar expression patterns. Upon further analysis, we noted that all three abiotic stresses increased the abundance of transcripts related to the JA metabolic pathway, which would contribute to the increased formation of the bioactive JA compound. Based on this knowledge, we infer that ethylene and JA have synergistically positive effects on resisting abiotic stresses. The ERF gene is an upstream component in both JA and ethylene signalling. The expression pattern of the ERF gene was also up-regulated, further supporting our hypothesis.

### Abiotic stresses modify the nod factor signalling pathways during early symbiotic nodulation

Early symbiotic nodulation involves four major developmental programmes: (1) the perception of nod factor, (2) the activation of calcium spiking, (3) root hair deformation and infection, and (4) early nodulin gene induction and nodule primordium formation. A comprehensive transcriptomic analysis of nodulation signalling pathway gene expression in response to abiotic stresses is lacking in the literature. We detected several transcripts for key genes involved in the nodulation signalling pathway. NFR genes are nod factor receptors and play a crucial role in the perception of nod factor. All three stresses increased the expression of the NFR genes. The DMI1 and DMI2 genes displayed similar expression patterns. In accordance with biotic stress, we hypothesise that all three abiotic stresses may enhance the perception of nod factor and the activation of calcium spiking. DMI3, which exhibits different expression patterns between abiotic stresses, may affect the perception of calcium spiking and deformation of root hairs. The expression of the DMI3 genes was down-regulated in the CS sample but up-regulated in the DS and SS stressed samples. The expression of the ENOD genes was down-regulated all together by the three stresses because the expression pattern of the ENOD transcriptional regulators ERN and NSP were altered by these stresses. NSP genes exhibiting decreased expression levels were predominantly due to the up-regulated RR genes. We hypothesise that NSP genes are targets of the RRs-dependent signalling pathway and that miR171h gene is crucial to regulate their expression in response to abiotic stresses and during early nodule development. Interestingly, the expression of the ENOD genes showed less of a decrease in the dehydration -stressed sample compared with the other two stressed samples, which may be due to the increased expression of ERN genes. Upon the above analyses, we concluded that all three stresses might negatively regulate the formation of nodule primordia.

### The role of auxin in root responses to cold stress

The cold-stressed root transcripts reported here were derived from our RNA-Seq data and thus reflect the expression of genes not only involved in a generalised root response to cold stress but also transcripts directly related to root development. We found that cold stress reduced lateral root formation, nodule number, and root length. In addition, we found that IAA application can increase the number of lateral roots and nodules, suggesting that the cold-induced decrease of lateral root formation and nodule number are dependent upon a reduction in auxin availability. AUX is an important auxin transporter. Our qTR-PCR results indicate that cold application appears to inhibit AUX expression. The expression of AUX increased after the application of exogenous IAA. We hypothesise that cold-induced reductions in auxin availability may depend on the decreased expression levels of AUX genes. Interestingly, IAA application is unable to increase the length of the main root. We hypothesise that the development of the main root requires the synergy of auxins and other hormones.

## Conclusions

To our knowledge, we here present the first whole transcriptome analysis of abiotic stresses using M. falcata and an in-depth RNA-seq analysis of its transcriptomes under optimal treatment conditions. Overall, these experiments and results have confirmed previously reported responses to abiotic stresses while addressing previously unknown details and fundamentally advancing our understanding of how modified gene expression patterns facilitate acclimation to abiotic stresses. We revealed the abiotic stress-responsive mechanisms underlying the metabolism and core signalling components of major phytohormones. In addition, we identified nod factor signalling pathways during early symbiotic nodulation that are modified by abiotic stresses.

## Methods

### Plant material

M. falcata seeds were obtained from the Agricultural Research Service (ARS) GRIN system (http://www.ars-grin.gov/), US Department of Agriculture (USDA). Seeds originating from Russia (accession number PI502449) were used for this work. Accession PI502449 is regarded as the most tolerant to winter injury [97] and was identified as diploid M. falcata [98]. Seeds of Medicago truncatula A17 and R108 were provided by BRC, UMR 1097, INRA, Montpellier, France. Seed treatments for germination have been published previously [97]. After germination, the plantlets were grown in a soil/vermiculite (3:1, v/v) mixture in a chamber incubated under controlled environment conditions (14-h photo-period, 60 μmol photons m^−2^ s^−1^, 22°/18 °C day/night regime, 70 % relative humidity).

### Evaluate the effect of cold, drought and salt stresses on M. truncatula 108-R, M. truncatula A17 and M.falcata PI502449

Plants grown for these assays were germinated as described above. After germination, 4-week-old plants grown in a soil/vermiculite (3:1, v/v) mixture at 24 °C under a 14-h photoperiod were used for abiotic stress treatments (14-h photo-period, 60 μmol photons m^−2^ s^−1^, 22°/18 °C day/night regime, 70 % relative humidity). For cold treatment, the plantlets were incubated at 0 °C (freezing chamber RuMED4001) for 6 h, 8 h, and 10 h under dim light (0.7-0.8 μmol sec^−1^ m^−2^). For drought treatment, the plants were grown without watering for 10 d, 12 d, and 14 d under dim light (0.7-0.8 μmol sec^−1^ m^−2^). For salinity treatment, the plants were irrigated with NaCl solution at 100 mM, 150 mM, and 200 mM for 10 d under dim light (0.7-0.8 μmol sec^−1^ m^−2^). After treatment, leaves and roots were collected respectively, and then measured the weight, mortality and soluble carbohydrates content. Three independent experiments were performed.

### Electrolyte leakage assays

Plants grown for electrolyte leakage assays were germinated as described above. After germination, 4-week-old plants grown in a soil/vermiculite (3:1, v/v) mixture at 24 °C under a 16-h photoperiod were used for abiotic stress treatments (14-h photo-period, 60 μmol photons m^−2^ s^−1^, 22°/18 °C day/night regime, 70 % relative humidity). For cold treatment, the plantlets were incubated at 0 °C (freezing chamber RuMED4001) for 2 h, 4 h, 6 h, 8 h, and 10 h under dim light (0.7-0.8 μmol sec^−1^ m^−2^). For dehydration treatment, the plantlets were transferred to dry Whatman 3 MM paper [4] for 0.5 h, 1 h, 1.5 h, and 2 h under dim light (0.7-0.8 μmol sec^−1^ m^−2^). For high salinity treatment, the plantlets were irrigated with NaCl solution at 200 mM, 400 mM, 600 mM, 800 mM, and 1 M for 2 h under dim light (0.7-0.8 μmol sec^−1^ m^−2^). After treatment, leaves were collected, and the electrolyte leakage was measured as previously described [97]. Three independent experiments were performed.

### Abiotic stress treatment for sequencing samples

Plants grown for abiotic stress treatment were germinated as described above. Dehydration, cold, and high salinity stress treatments were applied essentially as reported previously [99]. For dehydration treatment, plants were harvested from pots and desiccated on Whatman 3MM paper for 2 h at 24 °C under dim light (0.7-0.8 μmol sec^−1^ m^−2^). For cold stress, we transferred the plantlets to 0 °C (freezing chamber RuMED4001) for 8 h under dim light (0.7-0.8 μmol sec^−1^ m^−2^). For high salinity stress, we irrigated the plantlets with 1 M NaCl solution for 2 h under dim light (0.7-0.8 μmol sec^−1^ m^−2^). Plantlets without stress treatment were used as the standard condition, and all materials were stored in liquid nitrogen immediately after treatment and harvest. Two biological replicates per condition were used for further analysis. In every biological replicate, we mix five plantlets together.

### cDNA library preparation and sequencing

Total RNA from each sample was isolated using the TRIzol reagent (Invitrogen), and genomic DNA was digested using DNase (New England Biolabs). We used the OligoTex mRNA mini kit (Qiagen) to purify poly (A) mRNA from the total RNA. The protocol for cDNA synthesis has been previously described [100]. The mRNA was fragmented using an RNA fragmentation kit (Ambion). The first cDNA strand was synthesised using random hexamer primers, and the second cDNA strand was subsequently synthesised.

The sequencing library was constructed according to the manufacturer’s instructions (Illumina) for high-throughput sequencing. Fragments of approximately 300 nt were excised and enriched by 18 PCR cycles. The PCR products were sequenced using the Illumina HiSeq 2000 platform using101-cycle paired-end reads. Initial base calling and quality filtering of the Illumina GA-IIx image data were performed using the default parameters of the Illumina GA Pipeline GERALD stage (http://www.illumina.com). All sequencing reads were deposited into the Short Read Archive (SRA) of the National Center for Biotechnology Information (NCBI), and can be accessed under the accession number (SRR1956534, SRR1956535, SRR1956716, SRR1956718, SRR1956719, SRR1957050, SRR1956913, SRR1956721, SRR1982256, SRR1982261, SRR1982264 and SRR1982268).

### De novo transcriptome assembly

To obtain a better assembly result, two assembly methods (single k-mer (SK) method and multiple k-mer (MK) method) were used for the de novo assembly of these sequencing data. We first performed a test assembly using Illumina reads derived from the SC sample rep1 library with a series of k-mers (28–56) using ABySS [101]. To select an optimal k-mer parameter, we compared the summary statistics, including N50s, total contig number, and the total assembly size generated by each k-mer (Additional file 13). After careful consideration, k-mer = 41 was chosen for accuracy and the length of the assembled transcripts. Using these identified parameters, a total of 31,591,337,787 nt reads generated from all eight libraries ((dehydration, salt, cold and standard) × (two replicates)) were de novo assembled into contigs. Additionally, Trinity [102] has been shown to be the best SK assembler for transcriptome assembly [103]. Trinity was implemented with default parameters on our 8 library datasets. The results of the MK and SK were combined using the CAP3 programme with default parameters [104]. Finally, the redundant sequences were collapsed using the CD-HIT-EST algorithm [105], producing a total of 98,515 sequences. These sequences span 84,416,200 nt, with an average length of 857 nt. The assembly sequences were listed at (http://bioinformatics.cau.edu.cn/falcata/sequences/all.transcripts).

### Putative function, GO, and KEGG orthology annotations

The assembled reference sequences were aligned to NCBI nr database using BLASTx [106] with a threshold of e-value ≤1e^−6^, and the best three hits were chosen for putative function annotation. KAAS (KEGG Automatic Annotation Server) was used to identify biochemical pathways and to calculate the statistical significance of each pathway [107]. The GO annotations were obtained from scanning the transcriptome by using InterProScan [108].

### Differentially expressed transcripts

The sequencing reads of the four samples were mapped to the M. falcata transcriptome by SOAPaligner [109], with a maximum of three mismatches. Gene expression levels were calculated by RPKM. The expression difference was identified by DEGseq [110], in which Fisher’s exact test and the likelihood ratio were proposed to identify differentially expressed genes, and the P- and Q-values for each gene were calculated. Differentially expressed genes identified by DESeq were required to have a 2-fold change and P ≤ 0.05.

### qRT-PCR

We first selected six expressed and relatively stable genes displayed in the RNA-Seq data as internal control candidates. Two reference genes (ID: Mf94657 and Mf85797) were determined by geNorm [111] for qRT-PCR analysis. After the total RNA was extracted, reverse transcription was performed using M-MLV Reverse Transcriptase (Promega). The quantitative real-time PCR analysis was conducted using a CFX-96 Real-Time System (Bio-Rad) and SYBR Premix Ex Taq (TaKaRa). All of the specific primers used in this work are listed in Additional file 14.

### Determine ABA content

ABA content was measured as previously described [112]. For cold stress, we transferred the plantlets (4-week-old) to 0 °C (freezing chamber RuMED4001) for 0 h, 2 h, 4 h, 6 h under dim light (0.7-0.8 μmol sec-1 m-2). After cold stress, a total of 20 mg of homogenized M. falcata (PI 502449) and M. truncatula (A17) leaves were soaked in 1 ml of ABA extraction buffer (10 mM HCl and 1 % polyvinylpolypyrrolidone in methanol) and shaked at 4 °C for 16 h. After neutralization with 15 μl of 1 M NaOH, the supernatant was dried and resuspended in TBS. The ABA content was quantified using a Phytodetek ABA Test Kit (Agdia, USA). These experiments were repeated three times with similar results.

### Auxin treatment experiment

Plants grown for the auxin treatment experiment were germinated as described above, except that the plantlets were transferred to large Petri pots containing Fahraeus medium [113] vertically and inoculated with Sinorhizobium meliloti strain 1021 in Bergensen’s modified medium adjusted to an optical density at 600 nm [OD_600_] = 0.1. There were four plantlets per pot. Each pot was two-thirds covered with black paper on the outside. These pots were placed in a growth chamber at 24 °C under long days (16 h light, 8 h dark) for 72 h, and the pots were then divided into three sets. (a) Plantlets were supplemented with IAA to a final concentration of 10^−8^ M and grown at 4 °C under long days for 10 days before being transferred to 24 °C. (b) Plantlets were supplemented with IAA to a final concentration of 10^−8^ M and maintained at 24 °C under long days. (c) Plantlets were supplied with an equal amount of distilled water and maintained at 24 °C under long days. All of these plants were collected after 21 days for root measurements and RNA extraction.

## Additional files

Additional file 1:
**Figure showing effect of cold, drought and salt stresses on M. truncatula 108-R, M. truncatula A17 and M.falcata PI502449.** A, Relative raw weights (%) of root system and aerial part of three Medicago genotypes under different cold stress conditions. B, Relative raw weights (%) of root system and aerial part of three Medicago genotypes under different drought stress conditions. C, Relative content of solube carbohydrates of three Medicago genotypes under different salt stress conditions. D, Mortality of three Medicago genotypes under different cold stress conditions. (Student’s *t*-test, ***P* < 0.001, **P* < 0.01) (TIFF 13969 kb) Additional file 2:
**Figure showing electrolyte leakage of the DS, SS, and CS samples into leaf tissue.** Plants were germinated and treated as described in Methods. A, Electrolyte leakage in leaf tissue under cold stress. B, Electrolyte leakage in leaf tissue under dehydration stress. C, Electrolyte leakage in leaf tissue under high salinity stress. (Student’s *t*-test, ***P* < 0.001, **P* < 0.01) (TIFF 13168 kb) Additional file 3:
**Table listing statistics of raw sequencing reads.** (XLSX 9 kb) Additional file 4:
**Table listing summary of de novo assembly results.** (XLSX 8 kb) Additional file :5
**Table listing housekeeping genes.** These transcripts were identified as the most stably expressed across all conditions. (XLSX 102 kb) Additional file 6:
**Table listing differentially expressed transcripts potentially regulated by miRNAs.** (XLSX 91 kb) Additional file 7:
**Table listing transcripts differentially expressed between abiotic stress and standard.** (XLSX 1117 kb) Additional file 8:
**Figure showing heat map of expression profiles of transcription factor.** Expression, represented by Z scores, is shown for transcripts differentially expressed due to three abiotic stresses. Red indicates high expression, yellow indicates intermediate expression, and green indicates low expression. Transcripts have been grouped by TF family. (TIFF 21063 kb) Additional file 9:
**Showing effect of cold stress on ABA content in M. truncatula A17 and M.falcata PI502449.** A, ABA content in M. truncatula A17. B, ABA content in M. falcata PI502449. (Student’s *t*-test, ***P* < 0.001, **P* < 0.01) (TIFF 13100 kb) Additional file 10:
**Table listing differential expression genes involved in phytohormone metabolism and signal transduction.** (XLSX 11 kb) Additional file 11:
**Table listing differential expression genes involved in nod factor signaling pathways during early symbiotic nodulation.** (XLSX 9 kb) Additional file 12:
**Figure showing similarity and coverage of M. falcata transcripts to M. truncatula genes.** A, Histogram showing the frequency vs. percent similarity (positive amino acid identity) of M. falcata contigs to a M. truncatula peptide. B, Frequency vs. percent coverage (longest positive hit/peptide length) of M. falcata contigs to a *M. truncatula* peptide. (note: most assembled *M. falcata* transcripts have a high coverage, which significantly skews the histogram to the right) C, Smoothed colour density representation of the percent similarity (x-axis) of each *M. falcata* transcript plotted against the percent coverage of the *M. truncatula* protein similarity (y-axis). Plot produced using the ‘smoothScatter’ function in R, which produces a smoothed density representation of the scatter plot using a kernel density estimate (nbin = 100). Darker colour indicates a higher density of transcripts in a given position. (TIFF 2408 kb) Additional file 13:
**Figure showing optimisation of the** ***de novo*** **assembly.** Comparison of the total assembly size (y-axis, left) and the N50 (y-axis, right) using a variety of *k*-mers (x-axis). (TIFF 3922 kb) Additional file 14:
**Table listing primer sequences used for Real Time PCR.** (XLSX 13 kb)
