# Supplementary figures and images for: De novo transcriptome analysis of Medicago falcata reveals novel insights about the mechanisms underlying abiotic stress-responsive pathway
Source: BMC Genomics. 2015 Oct 19;16:818. doi: 10.1186/s12864-015-2019-x (PMC4615886; doi:10.1186/s12864-015-2019-x)

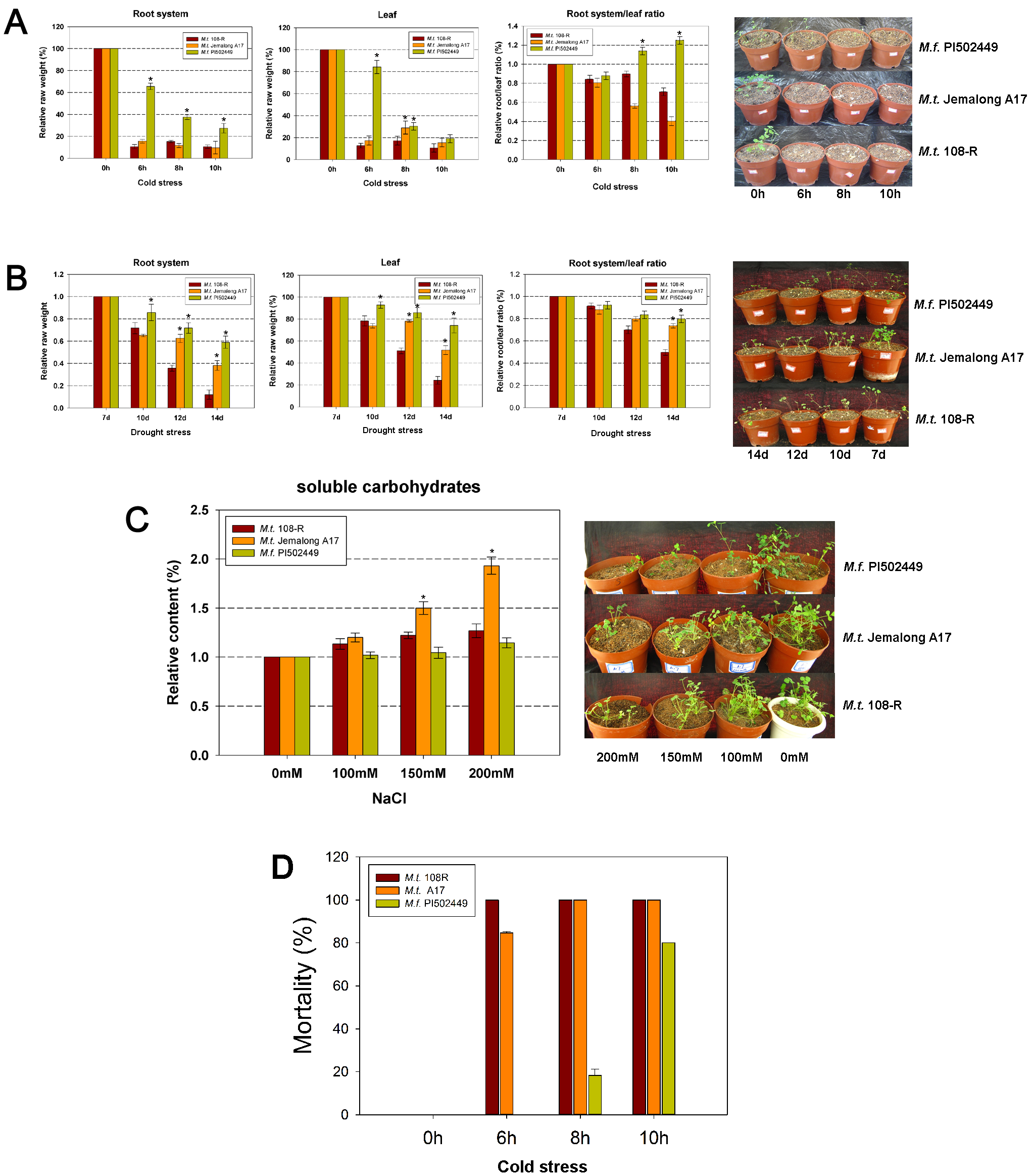

Supplement: Additional file 1: — Figure showing effect of cold, drought and salt stresses on M. truncatula 108-R, M. truncatula A17 and M.falcata PI502449. A, Relative raw weights (%) of root system and aerial part of three Medicago genotypes under different cold stress conditions. B, Relative raw weights (%) of root system and aerial part of three Medicago genotypes under different drought stress conditions. C, Relative content of solube carbohydrates of three Medicago genotypes under different salt stress conditions. D, Mortality of three Medicago genotypes under different cold stress conditions. (Student’s t-test, **P < 0.001, *P < 0.01) (TIFF 13969 kb) [file 12864_2015_2019_MOESM1_ESM.tif]

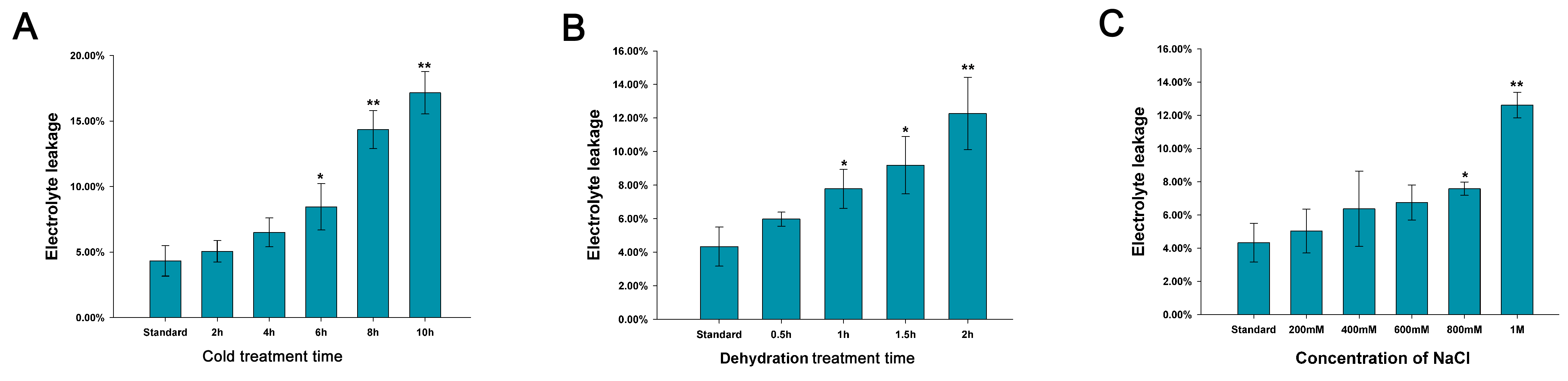

Supplement: Additional file 2: — Figure showing electrolyte leakage of the DS, SS, and CS samples into leaf tissue. Plants were germinated and treated as described in Methods. A, Electrolyte leakage in leaf tissue under cold stress. B, Electrolyte leakage in leaf tissue under dehydration stress. C, Electrolyte leakage in leaf tissue under high salinity stress. (Student’s t-test, **P < 0.001, *P < 0.01) (TIFF 13168 kb) [file 12864_2015_2019_MOESM2_ESM.tif]

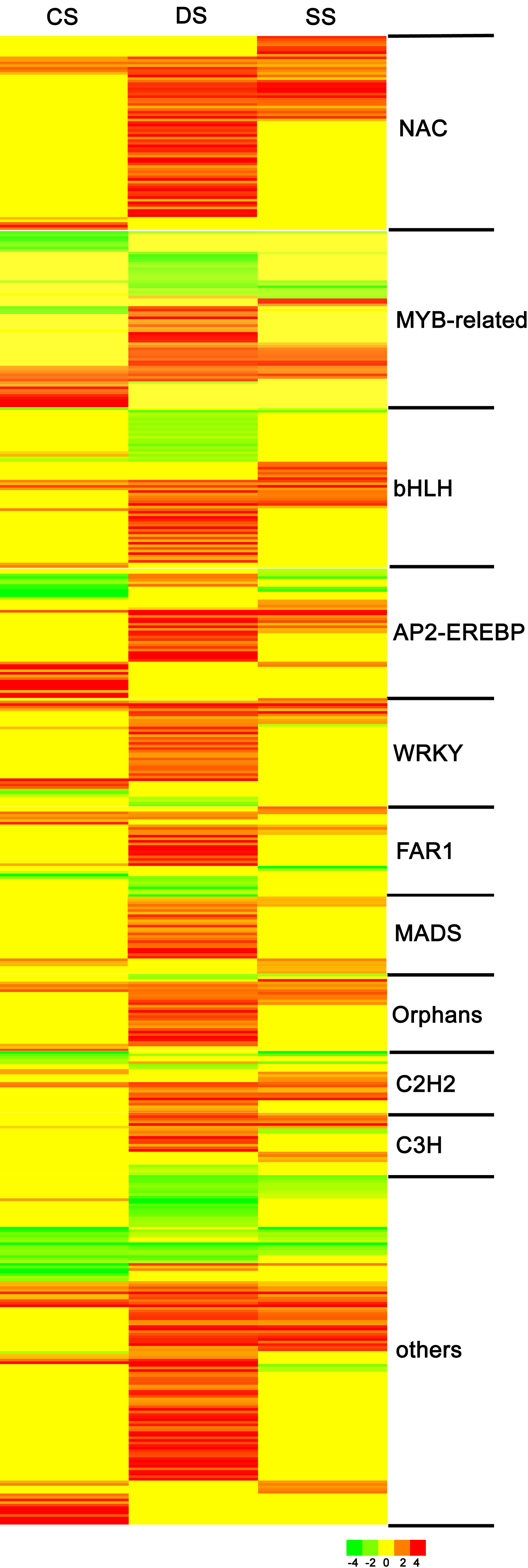

Supplement: Additional file 8: — Figure showing heat map of expression profiles of transcription factor. Expression, represented by Z scores, is shown for transcripts differentially expressed due to three abiotic stresses. Red indicates high expression, yellow indicates intermediate expression, and green indicates low expression. Transcripts have been grouped by TF family. (TIFF 21063 kb) [file 12864_2015_2019_MOESM8_ESM.tif]

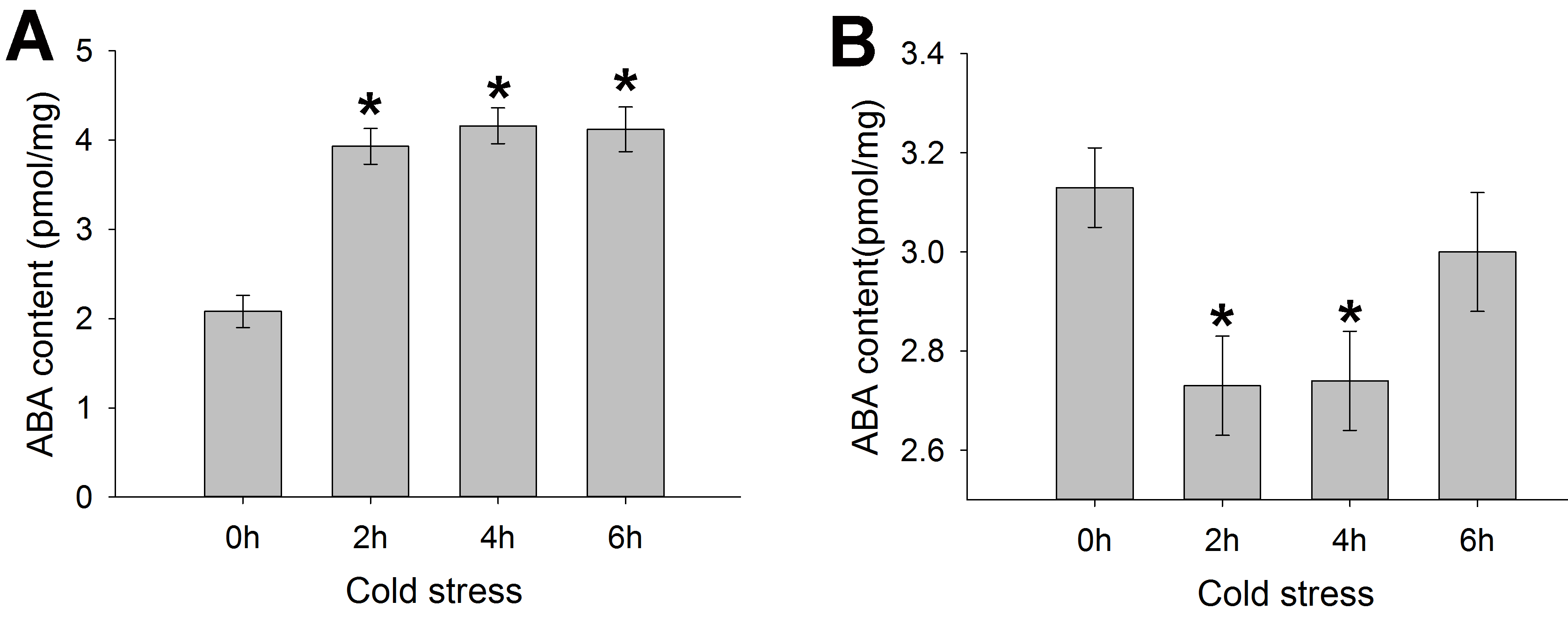

Supplement: Additional file 9: — Showing effect of cold stress on ABA content in M. truncatula A17 and M.falcata PI502449. A, ABA content in M. truncatula A17. B, ABA content in M. falcata PI502449. (Student’s t-test, **P < 0.001, *P < 0.01) (TIFF 13100 kb) [file 12864_2015_2019_MOESM9_ESM.tif]

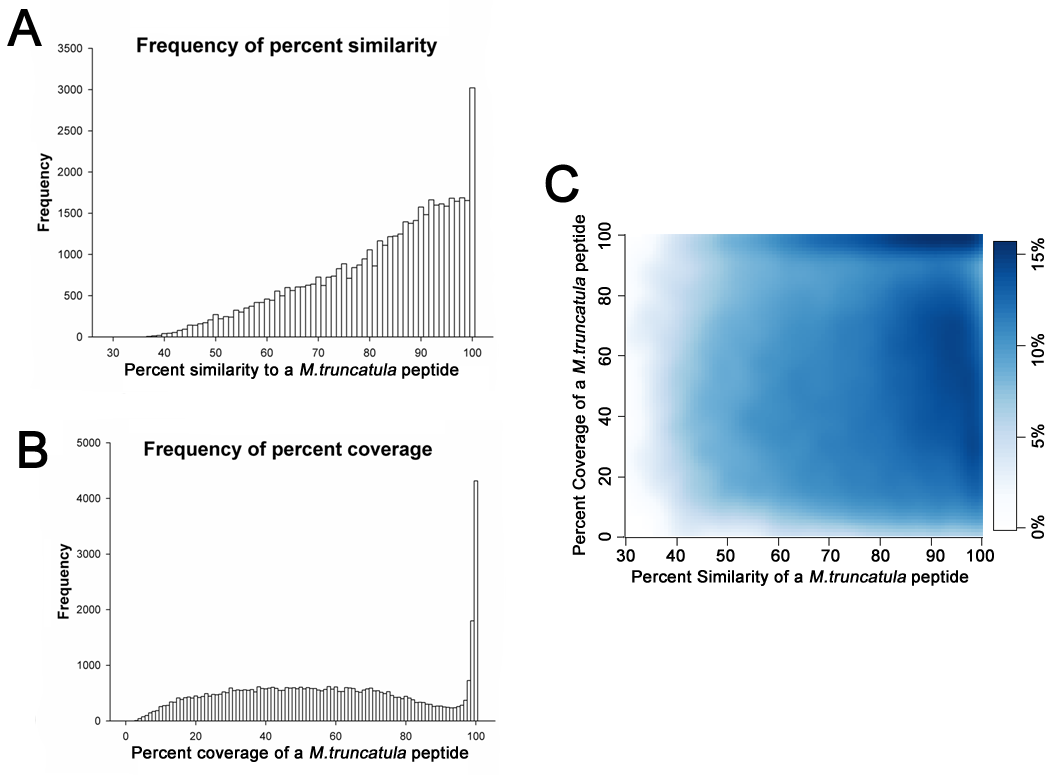

Supplement: Additional file 12: — Figure showing similarity and coverage of M. falcata transcripts to M. truncatula genes. A, Histogram showing the frequency vs. percent similarity (positive amino acid identity) of M. falcata contigs to a M. truncatula peptide. B, Frequency vs. percent coverage (longest positive hit/peptide length) of M. falcata contigs to a M. truncatula peptide. (note: most assembled M. falcata transcripts have a high coverage, which significantly skews the histogram to the right) C, Smoothed colour density representation of the percent similarity (x-axis) of each M. falcata transcript plotted against the percent coverage of the M. truncatula protein similarity (y-axis). Plot produced using the ‘smoothScatter’ function in R, which produces a smoothed density representation of the scatter plot using a kernel density estimate (nbin = 100). Darker colour indicates a higher density of transcripts in a given position. (TIFF 2408 kb) [file 12864_2015_2019_MOESM12_ESM.tif]
